# Supplementary material for: Where will it end? Pathways to care and catastrophic costs following negative TB evaluation in Uganda
Source: PLoS One. 2021 Jul 16;16(7):e0253927. doi: 10.1371/journal.pone.0253927 (PMC8284677; doi:10.1371/journal.pone.0253927)
Supplement: S1 Table — n = 51 for analysis (due to n = 51 known HIV status). OR = odds ratio; TB = tuberculosis. All variables found to be associated with accessing healthcare post-evaluation with a significance level of p≤0.1 in the univariate analysis were included in the multivariable analysis along with age and sex. A longer duration of symptoms after TB evaluation was associated with increased odds of attending healthcare facilities post-evaluation. Subjects living in rural settings and those with HIV infection had lower odds. Four or more healthcare attendances prior to TB evaluation trended towards association with increased odds of attendance post-evaluation but was non-significant. (PDF) [file pone.0253927.s001.pdf]

Variable		Adjusted OR	95%CI	Significance
Number of times healthcare facility visited prior to TB evaluation	0 – 3 times	1		0.06
	4+ times	5.16	0.96 – 27.76	
Living Environment	Urban/Peri-urban	1		0.02
	Rural	0.14	0.03 – 0.73	
Known HIV status	Negative	1		0.05
	Positive	0.19	0.04 – 1.00	
Symptom duration	/1 week increase in duration of symptoms	1.10	1.02 – 1.20	0.02
Age	/year of age	0.98	0.92 – 1.04	0.5
Sex	Female	1		0.2
	Male	0.30	0.06-1.65	
