# Supplementary material for: Where will it end? Pathways to care and catastrophic costs following negative TB evaluation in Uganda
Source: PLoS One. 2021 Jul 16;16(7):e0253927. doi: 10.1371/journal.pone.0253927 (PMC8284677; doi:10.1371/journal.pone.0253927)
Supplement: S2 Table — n = 51 for analysis (due to n = 51 known HIV status). OR = odds ratio; TB = tuberculosis; NTLP = National Tuberculosis and Leprosy Program. Variables that demonstrated an association with a significance level of p≤0.1 were taken through to multivariable analysis along with age and sex. (PDF) [file pone.0253927.s002.pdf]

Variable		OR	95%CI	Significance
Knowledge of negative TB test result	Not aware			0.9
	Aware	0.92	0.27 – 3.10	
Number of times healthcare facility visited prior to TB evaluation	0 – 3 times			0.05
	4+ times	3.24	1.00 – 10.49	
Test type	Smear Microscopy			0.2
	Xpert MTB/Rif	4.04	0.44 – 37.28	
Age	/year of age	0.98	0.95 – 1.02	0.4
Sex	Female			0.7
	Male	0.82	0.28-2.46	
Living Environment	Urban/Peri-urban			0.007
	Rural	0.19	0.05 – 0.64	
Following the NTLP algorithm	No			0.3
	Yes	1.94	0.55 – 6.88	
Mobile phone ownership	No			0.4
	Yes	1.69	0.54 – 5.34	
Known HIV status (n=51)	Negative			0.03
	Positive	0.27	0.08 – 0.88	
Income	≤Median income			0.5
	>Median income	0.69	0.23 – 2.06	
Symptom duration	/1 week increase in duration of symptoms	1.05	0.99 – 1.11	0.1
	No			0.7

Reported cause for symptoms found post-evaluation	Yes	0.78	0.26-2.34	
