# Supplementary material for: Where will it end? Pathways to care and catastrophic costs following negative TB evaluation in Uganda
Source: PLoS One. 2021 Jul 16;16(7):e0253927. doi: 10.1371/journal.pone.0253927 (PMC8284677; doi:10.1371/journal.pone.0253927)
Supplement: S3 Table — n = 51 for analysis (due to n = 51 known HIV status). TB = tuberculosis; USh = Ugandan Shillings; NTLP = National Tuberculosis and Leprosy Program. (PDF) [file pone.0253927.s003.pdf]

Variable		Total cost change (USh)	95%CI	Significance
Knowledge of negative TB test result	Not aware			0.9
	Aware	-90,900	-1,048,100 - 866,300	
Number of times healthcare facility visited prior to TB evaluation	0 – 3 times			0.2
	4+ times	517,750	-340,300 – 1,375,750	
Test type	Smear Microscopy			0.9
	Xpert MTB/Rif	-114,400	-1,475,400 – 1,246,550	
Age	/year of age	-4,300	-33,450 – 24,800	0.8
Sex	Female			0.4
	Male	434,950	-420,450 – 1,290,300	
Living Environment	Urban/Peri-urban			0.4
	Rural	-358,600	-1,219,300 – 502,050	
Following the NTLP algorithm	No			0.5
	Yes	357,350	-640,100 – 1,354,850	
Mobile phone ownership	No			1.0
	Yes	-23,450	-934,250 – 887,350	
Known HIV status (n=51)	Negative			1.0
	Positive	21,750	-873,700 – 917,200	
Pre-symptom household income	Change/10,000 USh increase in household income	7,400	850 – 13,951	0.03

Symptom duration	/1 week increase in duration of symptoms	16,850	-15,050 – 48,750	0.3
Reported cause for symptoms found post-evaluation	No			0.1
	Yes	-672,100	-1,514,000 – 169,750	
