# Supplementary material for: Where will it end? Pathways to care and catastrophic costs following negative TB evaluation in Uganda
Source: PLoS One. 2021 Jul 16;16(7):e0253927. doi: 10.1371/journal.pone.0253927 (PMC8284677; doi:10.1371/journal.pone.0253927)
Supplement: S1 File — This tool was adapted from a validated costing tool used in previous work by our group [11]. No questions related to financial expenditure were added or subtracted from the original costing tool. Additional questions were added to aid in discriminating different pathways to care used by study participants. (PDF) [file pone.0253927.s004.pdf]

MODIFIED COSTING TOOL FOR 'WHERE WILL IT END' STUDY V1.8 FINAL
Prepared by Thomas HA Samuels

Question/field	Q	Options
**Interview setup**		
(question for interviewer)<br>Interviewer initials	1	
(question for interviewer)<br>Date of the interview	2	Drop down calendar
(question for interviewer)<br>Generate new participant ID	3	Generate new ID number.
**Personal Details**		
What is your surname?	4	
And your first/Christian name?	5	
How old are you?	6	
What is your gender?	7	Male<br>Female
Which district do you live in?<br>Which county?<br>Which sub-county? (if relevant)<br>Which parish?<br>Which village?	8	District (free text)<br>County (free text)<br>Sub-county (free text)<br>Parish (free text)<br>Village (free text)
(Question for interviewer)<br>Is the participants address in an urban or rural location? If they have moved since testing, what location was it in at time of testing?	9	Rural<br>Urban
(Question for interviewer)<br>Is the patient alive or dead?	10	Alive<br>Dead<br><br>If answer is alive, complete rest of questionnaire. If answer is dead, answer questions labelled Ω then stop questionnaire
Do you own a mobile phone?	11	Yes<br>No

(If conducting phone interview, interviewer to answer yes without asking participant)		
If so, what is your mobile phone number?<br><br>(starting with 0; do not use +256)	12	To be displayed only if the answer to the previous question is yes
**TB testing**		
Before you had testing for TB, how many times did you seek health care or advice for your symptoms?	13	
And which of the following did you access?<br><br>For other and don't know, please specify the name or names of the facility:	14	Health centre I – II<br>Health centre III - IV<br>District Hospital<br>Referral Hospital<br>Local pharmacy/drugstore<br>Dispensary<br>Herbalist<br>Traditional practitioner<br>Private clinic or hospital<br>Community health worker<br>Other (specify free text)<br>Don't know (specify free text)
(Question for interviewer – using registry data)<br>In which of the following locations was the participant tested for TB?	15<br>Ω	Selected test centres (TBC)<br>1-<br>2-<br>3-<br>4-
(Question for interviewer – using registry data)<br>When was that test done?	16<br>Ω	Drop down calendar
(Question for interviewer – using registry data)<br>What method of TB testing was used?	17<br>Ω	GeneXpert<br>Smear microscopy
Were you told what the result of your TB test was?<br><br>(If no, and participant has not already been given results of testing, ask whether participant would like their results. If they agree, give the negative result.)	18	Yes<br>No
What symptoms did you have when you were tested?	19	Cough<br>Shortness of breath

If other, please specify:		Fever<br>Weight loss<br>Night sweats<br>Other (specify free text)
How long did your symptoms continue *after* TB testing? (in weeks)<br>(If less than a week, record as "1")	20	
Since your test, have you tested positive for, or been started on treatment for, TB?<br>(Can confirm on district registers)	21	No<br>Yes, not on treatment<br>Yes, on treatment
Did you know your HIV status at the time of your initial TB testing?	22	Yes<br>No
If so, what was your HIV status?	23<br>Ω	Field only to open if answer to previous question is Yes<br><br>Positive<br>Negative
**Healthcare sought after testing**		
Have you sought further healthcare or other advice about your symptoms since TB testing?	24	Yes<br>No
How many times have you sought advice since your negative test?<br><br>If the answer to the previous question is No, then enter answer "0".	25<br>ΔΔ	Answer to this question will determine how many repetitions of Questions 60 to 77 will be administered.
Did you have any further investigations done or treatment given by the clinic that did your TB test?	26	Yes<br>No
If yes, what were they?	27	To be displayed if answer to previous question is Yes<br><br>Chest X-ray<br>Examined by doctor<br>Antibiotics given<br>Referral made to another hospital
Have you found a cause for your symptoms?	28<br>^^	Yes<br>No
If yes, what was the cause?	29	Field only to open if answer to question 28 is Yes

And who gave you the answer?<br><br>For other and don't know, please specify name of facility:	30	Field only to open if answer to question 28 is yes:<br><br>Health centre I – II<br>Health centre III - IV<br>District Hospital<br>Referral Hospital<br>Local pharmacy/drugstore<br>Dispensary<br>Herbalist<br>Traditional practitioner<br>Private clinic or hospital<br>Community health worker<br>Other (specify free text)<br>Don't know (specify free text)
If no, what stopped you finding an answer?	31	Field only to open if answer to question 28 is no
**Income reported before developing symptoms**		
What was your primary employment, or normal work, before your symptoms started?<br><br>Please specify other:	32	School student<br>Technician (vocational training in automotives, health care, etc.)<br>Service (maid, driver etc.)<br>Factory worker<br>Farmer<br>Government employee (office admin)<br>Teacher<br>Retiree<br>Housewife/homemaker<br>Casual labourer<br>Security/Police/Guard<br>Unemployed<br>Other (specify free text)
If you were in paid work, how much do you estimate *your* average net wage from labour related activities (labour income), per month was before your symptoms started? (USh/month)	33	

If not in paid work or no income, enter "0"		
How many hours a week were you working before your symptoms started? (hours per week)<br><br>If not working, enter "0"	34	
How much do you estimate the average revenue from labour (income), after tax, of *your household* was per month, before your symptoms started? (USh/month)	35	
**Income changes and social consequences**		
What is your current primary employment, or normal work, or normal other main activity?<br><br>Please specify other:	36	School student<br>Technician (vocational training in automotives, health care, etc.)<br>Service (maid, driver etc.)<br>Factory worker<br>Farmer<br>Government employee (office admin)<br>Teacher<br>Retiree<br>Housewife/homemaker<br>Casual labourer<br>Security/Police/Guard<br>Unemployed<br>Other (specify free text)
If you are in paid work, how much do you currently estimate *your* average net wage from labour related activities (net labour income), per month is now? (USh/month) If not working, enter "0".	37	
How much do you estimate the average revenue from labour (net labour income), after tax, of *your household* is per month now? (USh/month)	38	
How many hours per week are you working now? (hours/week) If not working, enter "0".	39	
Approximately how many working days of income have you lost due to your symptoms after you were tested for TB? (days)	40	
Did you or your household receive any social welfare payment for health-related issues since the negative test for TB?	41	No<br>Yes

		If answer is no, skip to Question 46
Paid sick leave: amount (after tax) during the last month (USh/month)<br>If did not receive, enter "0"	42	
Disability grant: amount (after tax) during the last month (USh/month)<br><br>If did not receive, enter "0"	43	
Cash transfer for poor families: amount (after tax) during the last month (USh/month)<br><br>If did not receive, enter "0"	44	
Other cash transfer: amount (after tax) during the last month (USh/month)<br><br>If did not receive, enter "0"	45	
Did you or your household use any savings (cash or bank deposits) to cover costs due to your symptoms after your negative TB test?	46	Yes<br>No
If yes, how much did you use in total? (USh)	47	Field only to open if answer to previous question is yes
Did you borrow any money to cover costs due to your symptoms after your negative TB test?	48<br>*	Yes<br>No
If yes, how much did you borrow in total? (USh)	49	Field only to open if answer to previous question is yes
From whom did you borrow?<br>If other, please specify:	50	Field only to open if answer to question 48 is yes<br><br>Family<br>Neighbours/friends<br>Private bank<br>Cooperative employer<br>"Unofficial/Money lender" (Black Market)<br>Other (specify free text)
Are you expected to pay the loan back?	51	Field only to open if answer to question 48 is yes<br>Yes<br>No
Have you started paying back the loan?	52	Field only to open if answer to question 48 is yes

		Yes<br>No
What is the monthly repayment on the loan, including interest? (USh/month)	53	Field only to open if answer to question 48 is yes
Have you sold any property to finance the cost of your illness since your negative TB test?	54<br>**	Yes<br>No
If yes, what did you sell?	55	Field only to open if answer to question 54 is yes<br><br>Land<br>Livestock<br>Vehicle<br>Household item<br>Farm produce<br>Gold/jewellery<br>Other (specify free text)
How much money did you receive from the sale of all items of your property in total? (USh)	56	Field only to open if answer to question 54 is yes
Were the assets that you sold previously supporting the family income (or expenditure)?	57<br>***	Field only to open if answer to question 54 is yes<br>Yes<br>No
If yes, indicate monthly income previously generated by the assets: (USh/month)	58	Field only to open if answer to question 54 is yes and the answer to question 57 is yes
To what extent have the symptoms/illness affected the household financially?	59	No impact<br>Little impact<br>Moderate impact<br>Serious impact<br>Very serious impact
**Review of visits to facilities after TB testing**<br>The following set of questions, shaded grey, need to be repeated a number of times defined by the answer to question 25 (marked ΔΔ).		Each successive round will be labelled by sequential letters (a-z)
For this visit to another facility for care or advice for your symptoms, what type of facility did you visit?<br><br>If other or unknown, please enter the name of the facility.	60a	Health centre I – II<br>Health centre III - IV<br>District Hospital<br>Referral Hospital

		Local pharmacy/drugstore<br>Dispensary<br>Herbalist<br>Traditional practitioner<br>Private clinic or hospital<br>Community health worker<br>Other (specify free text)<br>Don't know (specify free text)
How many weeks after TB testing did you visit this provider? (weeks after)	61a	
How much *total travel time* to and from the facility did you spend visiting this provider?	62a	
How much *total time* did you spend visiting this provider? Include waiting times, consultations, waiting for any lab results, etc.	63a	
Did you spend any out-of-pocket money on medical expenses including consultation fees, testing, medicines, or other items such as nutritional supplements?	64a	Yes<br>No
What is the TOTAL amount of ALL money and goods (including all things that were borrowed, sold or traded) that you and any other people in your household plus any guardians or caregivers had to use, that you or they would not have had to if you never had these symptoms or had to visit this provider? (USh)	65a	
How much out-of-pocket money did you spend during this visit for:<br>DAY CHARGES?<br>(Fees for hospital days. Only for hospitalisations, and only to be filled if not covered by the cost items below (consultation fee etc.)). If participant was never hospitalised, write "0". (USh)	66a	
How much out-of-pocket money did you spend during this visit for:<br>CONSULTATION FEES?<br>(Other charges, not covered under day charge, including direct payments to health care staff) (USh)	67a	
How much out-of-pocket money did you spend during this visit for:	68a	

RADIOGRAPHY AND OTHER IMAGING?<br>(out-of-pocket payments for imaging investigation (x-rays, CT-scan, ultrasound), TB-specific and other) (USh)		
How much out-of-pocket money did you spend during this visit for:<br>LAB TESTS?<br>(out-of-pocket payments for all tests) (USh)	69a	
How much out-of-pocket money did you spend during this visit for:<br>OTHER PROCEDURES?<br>(out-of-pocket payments for biopsy, bronchial lavage, other surgery related to symptoms etc.)	70a	
How much out-of-pocket money did you spend during this visit for:<br>MEDICINES?<br>(Any medicine prescribed for the participants symptoms)	71a	
How much out-of-pocket money did you spend during this visit for:<br>ANY OTHER MEDICAL PAYMENTS?	72a	
How much out-of-pocket money did you spend during this visit for:<br>TRAVEL?<br>(out-of-pocket payments for travel to facility (does not include income loss), for both participant and any household member)	73a	
How much out-of-pocket money did you spend during this visit for:<br>FOOD?<br>(out-of-pocket payment for addition food bought in relation to travelling to the health care visit, and during visit or hospitalisation, for both participant and any household member)	74a	
How much out-of-pocket money did you spend during this visit for:<br>OTHER EXPENSES INCLUDING ACCOMMODATION?<br>(includes out-of-pocket payments related to rent a room/bed during health care visits, and any other non-medical payments related to health care visit, for both participant and any household member).	75a	
Did you get any health insurance reimbursement for this visit? If so how much?	76a	

(amount reimbursed to participant through medical insurance (private or public scheme) so far, does not include expected future reimbursement)<br><br>If participant is not insured, write "0"		
How much money did you lose by not working and instead attending this facility?<br>(if the participant is salaried, or if the participant is unemployed, this may be "0")	77a	
**For interviewer**		
Did the participant complete the survey?	78<br>¥	Yes<br>No
If no, what question did they stop at?	79	Field only to open if answer to previous question is no.
If no, why not?	80	Field only to open if answer to question 78 is no.<br><br>Had to leave early<br>Survey was too long<br>Issues with survey content<br>Other (specify free text)
