# Supplementary material for: Where will it end? Pathways to care and catastrophic costs following negative TB evaluation in Uganda
Source: PLoS One. 2021 Jul 16;16(7):e0253927. doi: 10.1371/journal.pone.0253927 (PMC8284677; doi:10.1371/journal.pone.0253927)
Supplement: Attachment — Submitted filename: Response to the reviewers WWIE v1.0 20210511.docx [file pone.0253927.s006.docx]

London School of Hygiene and Tropical Medicine,

Keppel Street,

Bloomsbury,

London. WC1E 7HT

United Kingdom

12.05.2021

Dear Editor of PLOS One,

It is with pleasure that we are submitting our revised article to PLOS One entitled:

**Where will it end? Pathways to care and catastrophic costs following negative TB evaluation in Uganda.**

Manuscript ID number: PONE-D-21-07641

We would like to thank the reviewers for their valuable time and useful contributions to the manuscript. We have carefully considered and addressed each of the comments provided and have made revisions in line with their recommendations. Herein, we are presenting a more comprehensive and improved manuscript for publication.

Point-by-point responses to each of the reviewers’ comments are included within the revised submission. We thank you for considering our revised manuscript for publication and look forward to your reply.

Yours sincerely,

Thomas Samuels

Hospital for Tropical Diseases

University College London Hospitals NHS Foundation Trust

[thasamuels@gmail.com](mailto:thasamuels@gmail.com)

[thomas.samuels@nhs.net](mailto:thomas.samuels@nhs.net)

T +44 (0) 203 456 7890

M +44 (0) 7734 111 713

| Reviewer | Point | Line | Reviewer Comment | Response |
| --- | --- | --- | --- | --- |
| 1 | 1 | 87-88 | One community-level TB microscopy Centre was selected per area” can this be described further how the selection was done for instance random, systematically, convenience etc. | The centres were selected from amongst those used in a trial to assess the efficacy of on-site GeneXpert testing in local community health centres in Uganda - https://doi.org/10.1186/s13012-020-00988-y  One centre was selected at random from each of the four pre-defined areas from those participating in the above trial. These areas were selected *a priori* to illustrate a wider variety of patient experience.  We have amended the methods section to make this clearer |
| 1 | 2 | Figure 1 | In figure 1, is it possible to key in the numbers in the participant tracing procedure to show how you ended up with the numbers you surveyed. In the same figure please give the key for the abbreviations ‘NOK’ and ‘VHT’ | Unfortunately, we do not have any more granular data than whether participants were traced by phone, in person, or not at all. This data is available from Table 1. We agree with the authors points about the abbreviations and have amended the legend of the figure. |
| 1 | 3 | Table 1 | In Table 1, please state the denominator for the proportions calculated and the units for age. | We agree and have made the changes suggested by the reviewer. |
| 1 | 4 | 158-160 | In line 158-160, the six participants who later turned to be positive and were on anti-TB drugs, how did you handle their data?  (i) Were they part of the 31 who visited health care post-evaluation? (ii) If they were part of the 31, won’t their costs be different from the others who were not on anti-TB drugs? | In response to the reviewer:   1) They were part of the 31. 2) If diagnosed with TB, the subsequent healthcare visits after the visit in which that diagnosis was made were not included in this analysis as for our purposes, they constituted a completed healthcare pathway. TB medication itself is free in Uganda as it is provided by the state, but analysis beyond the point of diagnosis would have included further costs from clinic visits etc. that went beyond the scope of the study. |
| 1 | 5 | Figure 4, 208-209 | In figure 4, in line 208-209 you have stated what the slices represented, which data did you use to calculate the proportions? One can easily get mixed up with proportions described in line 194 to 199. | In figure 4, all direct costs incurred by participants were summed, and then broken down by the type of expenditure as shown. The fraction of overall total costs represented by each type of expenditure was then calculated and is presented as the pie chart in figure 4.  We agree with the reviewer that the reference to Figure 4 in the main text is misleading in this regard as it refers to different data. We have corrected this. |
| 1 | 6 | 330 and 372 | In line 330, reference 7 and line 372, reference 21 they appear to be incomplete. Are they journal articles? not clear what type of references they are. | It appears our referencing software had not fully completed these two references. We have corrected the issue and thank the reviewer for bringing this to our attention. |
| 1 | 7 | Discussion | One of the discussion points, given that majority of those that visited health facilities, visited private health facilities could it explain the high direct medical costs per facility. | The reviewer makes an excellent point. In data that we have not reported in the manuscript, we found that visits to private facilities had a higher median cost than state facilities (59,000Ush for private facilities vs. 37,000Ush for a district hospital and 27,000Ush for a local health centre). However, perhaps due to the small number of participants in the study, this difference was not statistically significant. Other studies done similar populations tend to support this point.  This is unlikely to only factor at play, however. In Ugandan state-funded health facilities, medical services beyond a simple consultation, such as radiographs and medications, are often not free at the point of use and health insurance is non-existent. The reported direct medical costs are therefore likely due to more than just the type of facility attended and likely reflect the generally high out-of-pocket expenditure required in this health system.  We chose not to include these points within the discussion as there were other things we wished to explore in more detail. |
| 1 | 8 | Conclusions | A suggestion in your recommendations in the conclusion: It would be helpful to do a comparative study between the costs encountered by those having positive TB evaluation and negative TB evaluation in the same population to know the magnitude in both groups. | We completely agree with the reviewer and have added this suggestion to the conclusion. |
| 2 | 1 |  | Authors have mentioned it in the limitation, but collecting information on cost incurred over past 9-10 months may have serious recall issues. Have authors tried to access some documents to verify at least the resource use? | We agree with the reviewer that these sorts of documents would assist in validating some of the cost results to some extent. Unfortunately, in the vast majority of cases very few such documents exist, and we were not able to collect any within the limits of the study. Ideally, further prospective research needs to occur to validate cost data, as we suggest in the conclusion. |
| 2 | 2 |  | I would like see more details of indirect cost estimation. How was it estimated if someone was not employed? Was it just by asking how much income they lost for healthcare faculty visit? | Indirect cost estimation was calculated as the fraction of annual reported income lost based on the number of days of work reported lost due to symptoms after TB evaluation/number of total work-days per annum. Separate data was collected asking participants how much lost income occurred due to individual healthcare visits. This was not added to the total calculated above but reported separately as per the results section. If a participant was not employed (almost always due to subsistence farming being their primary economic activity), they were asked to estimate the cost of their lost time. We also attempted to capture the economic costs to these participants by reporting dissavings in the results.  We have amended the methods to further detail how indirect cost data was calculated |
| 2 | 3 | Figure 2 | Figure 2 may not be required and can be explained in text only. | We agree with the reviewer that Figure 2 can be explained in text alone. However, we feel that the simplicity and clarity of the figure is a strength. Given that it remains within the overall figure limit of the paper, we would ideally prefer to keep it in the manuscript. |
